# The applicability of markerless motion capture for clinical gait analysis in children with cerebral palsy

**DOI:** 10.1038/s41598-024-62119-7

**Published:** 2024-05-24

**Authors:** Koen Wishaupt, Wouter Schallig, Marleen H. van Dorst, Annemieke I. Buizer, Marjolein M. van der Krogt

**Affiliations:** 1 Department of Rehabilitation Medicine, Amsterdam UMC location Vrije Universiteit Amsterdam, Amsterdam, The Netherlands; 2 Amsterdam Movement Sciences, Rehabilitation & Development, Amsterdam, The Netherlands; 3 Emma Children’s Hospital, Amsterdam UMC, Amsterdam, The Netherlands

**Keywords:** Movement disorders, Paediatric research

## Abstract

The aim of this comparative, cross-sectional study was to determine whether markerless motion capture can track deviating gait patterns in children with cerebral palsy (CP) to a similar extent as marker-based motion capturing. Clinical gait analysis (CGA) was performed for 30 children with spastic CP and 15 typically developing (TD) children. Marker data were processed with the Human Body Model and video files with Theia3D markerless software, to calculate joint angles for both systems. Statistical parametric mapping paired t-tests were used to compare the trunk, pelvis, hip, knee and ankle joint angles, for both TD and CP, as well as for the deviation from the norm in the CP group. Individual differences were quantified using mean absolute differences. Markerless motion capture was able to track frontal plane angles and sagittal plane knee and ankle angles well, but individual deviations in pelvic tilt and transverse hip rotation as present in CP were not captured by the system. Markerless motion capture is a promising new method for CGA in children with CP, but requires improvement to better capture several clinically relevant deviations especially in pelvic tilt and transverse hip rotation.

## Introduction

Cerebral palsy (CP) is the most common cause of childhood motor disability, affecting approximately 1 in every 500 new-born children^1–3^. Many children with CP have walking problems that are due to motor impairments such as spasticity, contractures, or weakness^1,4^. Clinical gait analysis (CGA) is often performed to provide guidance on which impairments to target with medical and rehabilitation interventions^5,6^. For CGA joint kinematics and kinetics, either with or without electromyography, are measured and compared to normative reference data to determine underlying motor impairments and guide treatment planning^7,8^.

3D marker-based motion capture has long been considered the standard in CGA^9^. However, it has several practical limitations that impact its application in clinical use^10–12^. Marker-based CGA requires highly skilled personnel to place retroreflective, skin-mounted markers on anatomical landmarks^12^, according to a specific marker model (e.g. Human Body Model^13^, Helen Hayes Model^14^, or Calibrated Anatomical System Technique^15^). This preparation phase is time-consuming and requires the child sustain a standing position for a considerable amount of time, which can be challenging for children with CP. Additionally, marker placement makes wearing minimal, skin-tight clothing mandatory and the removal of markers can be experienced as uncomfortable. Furthermore, measurement errors like soft tissue artifacts and inconsistent marker placement are inherent to marker-based CGA^16–18^.

Markerless motion capture is an emerging technique in the field of gait analysis^19^ and takes away the need for skin-mounted markers and some of the accompanying practical limitations. This method uses less costly (than optical cameras), high-definition 2D cameras and trained neural networks to identify 2D positions of body landmarks in every video frame individually^20^. An articulated multi-body model is scaled to fit the subject-specific landmark positions in 3D space, and an inverse kinematic approach is used to estimate the 3D pose of the subject^21^. One of those markerless motion capture solutions is Theia3D (Theia Markerless Inc., Kingston, ON, Canada). The inter-session variability is lower when using this software compared to marker-based measurements, as inconsistencies in marker placement do not affect the repeatability of the measurements^22^. However, markerless motion capture may constrain reality to fit its underlying model more than marker-based models, which could lead to improved inter-session variability at the cost of specificity.

Despite the methodological benefits a markerless solution has to offer, it is unknown whether markerless motion capture is capable of accurately tracking deviated gait patterns as seen in children with CP. Previous studies have shown promising results in able-bodied adults assessing gait kinematics in the sagittal and frontal plane using markerless motion capture^21^. However, markerless motion capture showed dissimilarities in transverse plane kinematics compared to marker-based motion capture^21,23^. No studies have compared marker-based and markerless motion capture in typically developing children. So far, only a few studies have evaluated markerless motion capture in clinical populations^24–26^, including small sample sizes of children with CP^24,25^. These studies evaluated the system on a group level. However, in children with CP large individual differences are observed. Therefore, the potential of markerless motion capture to measure deviated gait patterns present in children with CP requires more attention, especially because rotations in the transverse plane are often increased in children with CP^8,27^. In other words, it is unknown if the specificity of the markerless motion capture is good enough for capturing CP gait, despite its lower inter-session variability. Furthermore, when interpreting CGA outcomes, patient data is typically compared to a reference data set of unaffected individuals. For proper clinical implementation, rather than to look at absolute differences between systems, it is therefore important that the markerless system is also capable of detecting clinically relevant gait deviations with respect to normative reference data to a similar extent as the marker-based system. The present study compares gait kinematics obtained with a markerless system (Theia3D) and a marker-based system (Human Body Model (HBM)) for CGA of TD children and children with CP on both group- and individual level. Additionally, gait deviations in CP relative to TD are compared between systems.

## Methods

### Study design and participants

A comparative cross-sectional research design was employed. Fifteen typically developing (TD) children were included (9 girls, age: 11.9 ± 4.0 years, height: 149.4 ± 22.5 cm, weight: 42.9 ± 16.2 kg). Furthermore, 30 children with spastic CP (9 girls, age: 11.9 ± 4.2 years, body height: 146.5 ± 22.2 cm, body weight: 38.3 ± 15.3 kg, Gross Motor Function Classification System (GMFCS) level: I: 13, II: 15, III: 2) were included, as part of their standard clinical care. All children (if age > 12 years) and their parents (if age < 16 years) gave written informed consent for their data to be used for research purposes. The requirement for medical ethical review of this study was waived by the local medical ethics committee of the Amsterdam UMC under the Medical Research Involving Human Subjects Act in the Netherlands (#2023.0332). All methods were carried out in accordance with these guidelines and regulations and conformed to the Declaration of Helsinki guidelines.

### Procedures

**Figure 1:**
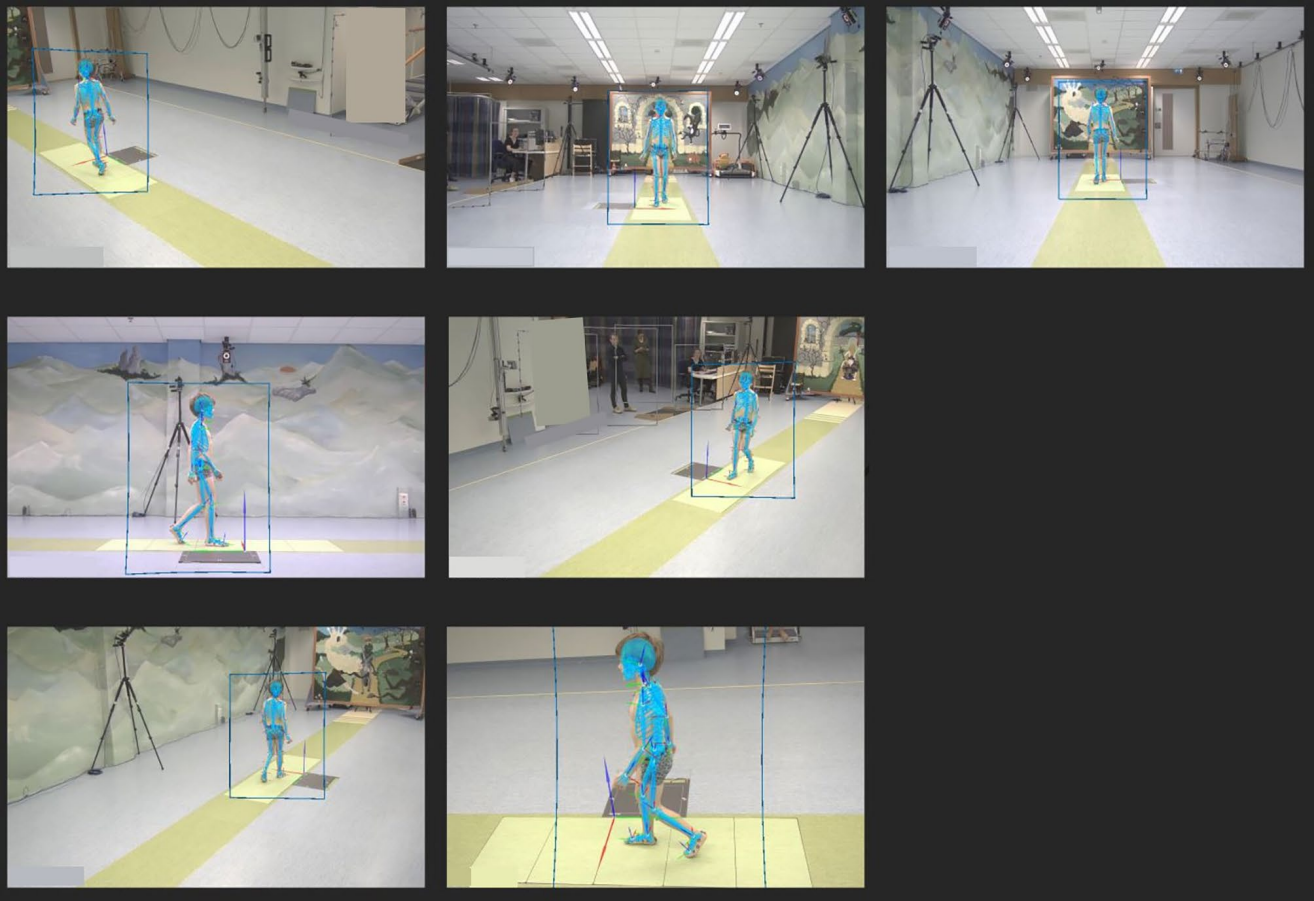
Experimental set-up, showing the viewpoint of all 7 video cameras with the Theia3D overlay.

The markerless camera system (7 Blackfly S USB3 cameras, Teledyne FLIR LLC, Wilsonville Oregon, USA; resolution: 1920 × 1200, 2.3 megapixels, focal length: 6.5–15.5 mm), as well as the marker-based camera system (4 Vicon Vantage V5 cameras + 8 Vicon T40S, Vicon Motion Systems Ltd., Oxford, United Kingdom), were positioned at a distance of 2 to 3 m around a 10-m walkway (Fig. 1). Both systems were connected to Vicon Nexus software (version 2.14, Vicon Motion Systems Ltd., Oxford, United Kingdom) to time- and spatially synchronize system calibration and data collection. Calibration procedures were performed prior to each measurement day and resulted in the same global reference frame for both systems.

All children wore minimal skin-tight clothing and no footwear. 26 retroreflective markers were placed according to the HBM protocol^13,28^. For each child, a static calibration trial was conducted for the marker-based system. No calibration trial was required for the markerless system. Children were instructed to walk at comfortable walking speed along the walkway, until at least five successful strides were captured. Both video data and marker data were collected at a frequency of 100 Hz.

### Data analysis

Marker-based data were processed using Vicon Nexus software (version 2.14, Vicon Motion Systems Ltd., Oxford, United Kingdom). Data were labelled and missing markers were filled using a Woltring filter (maximum gap length 10) and the gap-filling tools provided by Vicon Nexus (rigid body fill, pattern fill, cyclic fill). The labelled marker data were imported into the Gait Offline Analysis Tool (GOAT, version 4.2, Motek Medical B.V., Houten, The Netherlands). In GOAT the data were filtered using a built-in second-order low-pass Butterworth filter (6 Hz) and time-normalized per gait cycle^29^. Kinematic curves of the lower extremity and trunk were calculated following HBM^13^. This model constrains the trunk to have three degrees of freedom (DOFs), while allowing six DOFs in the pelvis, three DOFs in the hip, one DOF in the knee, and two DOFs in the ankle.

For the markerless processing, video data were transcoded using a codec (h.264) in the Vicon Nexus software. The transcoded video files were processed (default settings, 6 Hz low-pass GVCSPL filter) using the Theia3D software (v2023.1.0.3160, Theia Markerless Inc., Kingston Ontario, Canada). The Theia3D default inverse kinematic model was used, which has six DOFs in the pelvis and three DOFs in the hip, knee and ankle^21^. The Theia3D output, i.e. 4 × 4 transformation matrices of each body segment, were input in Vicon ProCalc (version 1.6.0, Vicon Motion Systems Ltd., Oxford, United Kingdom) to calculate the segment and joint angles of the lower extremity and trunk, for the same gait cycles as the marker-based data.

Five gait cycles from the right leg in TD children and the most affected leg in children with CP were analysed. If both legs were equally affected, five right gait cycles were selected. Kinematics of the trunk, pelvis, hip joint, knee joint, ankle joint (only sagittal plane), and foot progression were assessed. Frontal and transverse plane kinematics of the knee were fixed horizontal lines due to joint constraints in the HBM model. For the ankle, only the sagittal plane motion was comparable between systems due to disparities in joint definitions, where Theia3D has 3 DOF ankle, while HBM has a 2 DOF ankle with one similar sagittal axis and a second pronation/supination axis. This pronation/supination axis is thereby not aligned with the foot coordinate axes, and therefore not comparable between systems^30^. In Matlab (R2017b, The MathWorks Inc, Natick, Massachusetts) the markerless data were filtered with a second-order low-pass Butterworth filter (6 Hz), resulting in the same phase shift in both marker-based and markerless data post-filtering. The mean of every kinematic variable was calculated over the five gait cycles per child for both measurement systems and used for further analysis. For both TD children and children with CP, the group mean and standard deviation (SD) were calculated per kinematic variable for both the marker-based and markerless system. The mean TD data was also used as the normative reference group ('norm'). The deviation from the norm data was calculated to correct for differences in underlying model definitions between the two systems and to identify differences in patient data from a reference group per system, as is generally done in clinical gait analysis. For each stride in each child with CP, the angular difference was calculated from the group average of TD children (which served as reference data) and subsequently averaged per measurement system.

### Statistical analysis

The differences between the motion capture systems were assessed using Statistical Parametric Mapping (SPM) paired sampled t-tests for each kinematic variable. For all three data types, i.e. TD children, children with CP, and deviations from the norm, the individual means (averaged over five gait cycles) were used as input for the SPM analysis. The root mean square errors (RMSE) for the significantly different parts of the kinematic curves between systems were calculated. To quantify inter-subject variability for each group, the SD was averaged over the gait cycle for both TD children and children with CP. To assess individual subject-level differences between measurement systems, mean absolute deviations (MAD) for the full kinematic curves were determined per child and averaged across all children. The significance level was set at < 0.05 for all tests. Differences were considered clinically relevant when RMSE exceeded 5°^31^.

## Results

**Figure 2:**
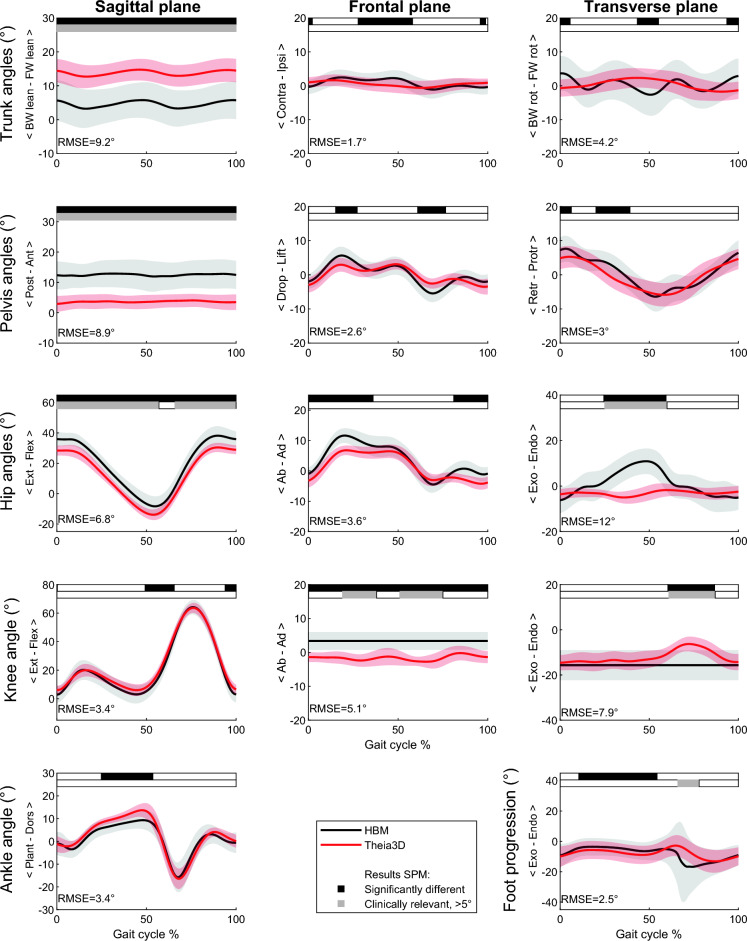
Typically-developing children—kinematic output (group mean ± SD) of the marker-based (HBM) and markerless (Theia3D) measurement systems. SPM outputs are displayed in the bars above each figure (white = non-significant, black = significant, grey = clinically relevant difference, > 5°). The RMSE of the significantly different parts of the curves are presented in the bottom left corner of each subplot.

For TD (Fig. 2) systematic offsets > 5° were observed in the sagittal plane over the whole gait cycle, with the markerless system showing more anterior tilt in the trunk, posterior tilt in the pelvis and flexion in the hip (RMSE of 9.2°, 8.9° and 6.8° respectively). One other significant and clinically relevant difference was found in the transverse hip rotation from mid-stance until toe off, showing less endorotation for the markerless system (RMSE of 12.5°). Furthermore, differences were observed in the frontal and transverse knee rotations (RMSE of 5.1° and 7.9°, respectively).

**Figure 3:**
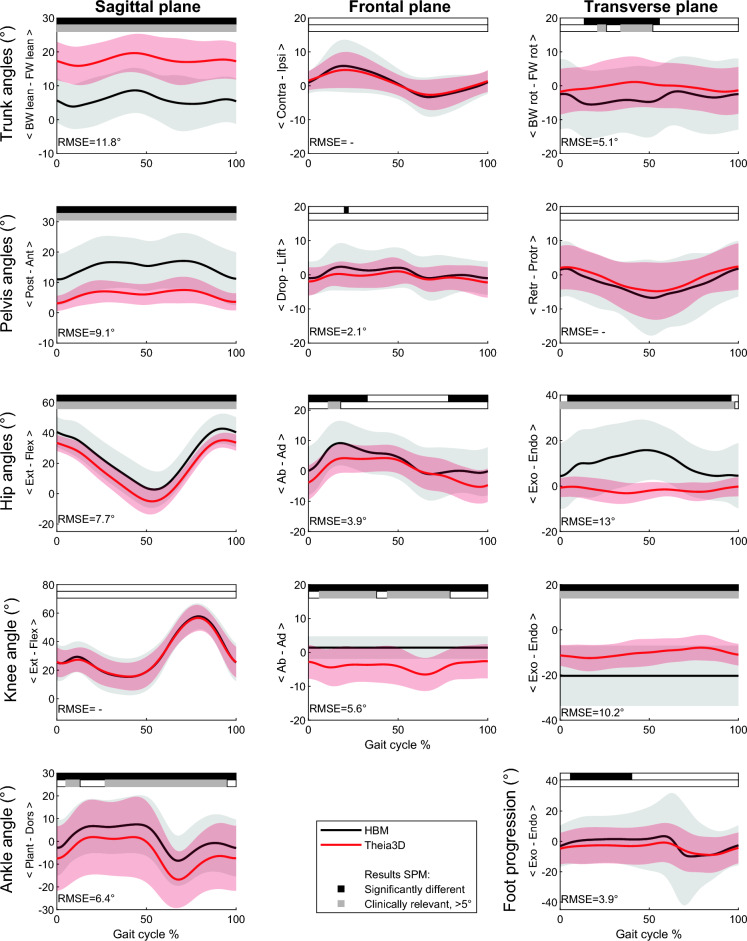
Children with CP—kinematic output (group mean ± SD) of the marker-based (HBM) and markerless (Theia3D) measurement systems. SPM outputs are displayed in the bars above each figure (white = non-significant, black = significant, grey = clinically relevant difference, > 5°). The RMSE of the significantly different parts of the curves are presented in the bottom left corner of each subplot.

In CP (Fig. 3), generally similar differences were found as for TD. A slightly higher offset than in TD was found in the sagittal plane, for trunk, pelvis and hip movement, with an RMSE of 11.8°, 9.1°, 7.7° respectively. Furthermore, less endorotation was found for the hip over the whole gait cycle and less trunk rotation in early and late stance for the markerless system, with an RMSE of 13.0° and 5.1° respectively. Knee frontal and transverse rotations were again different between the two systems, with an RMSE of 5.6° and 10.2°, respectively. In addition to TD the sagittal ankle angle was systematically in more plantarflexion for the markerless system over the whole gait cycle, with an RMSE of 6.4°.

**Figure 4:**
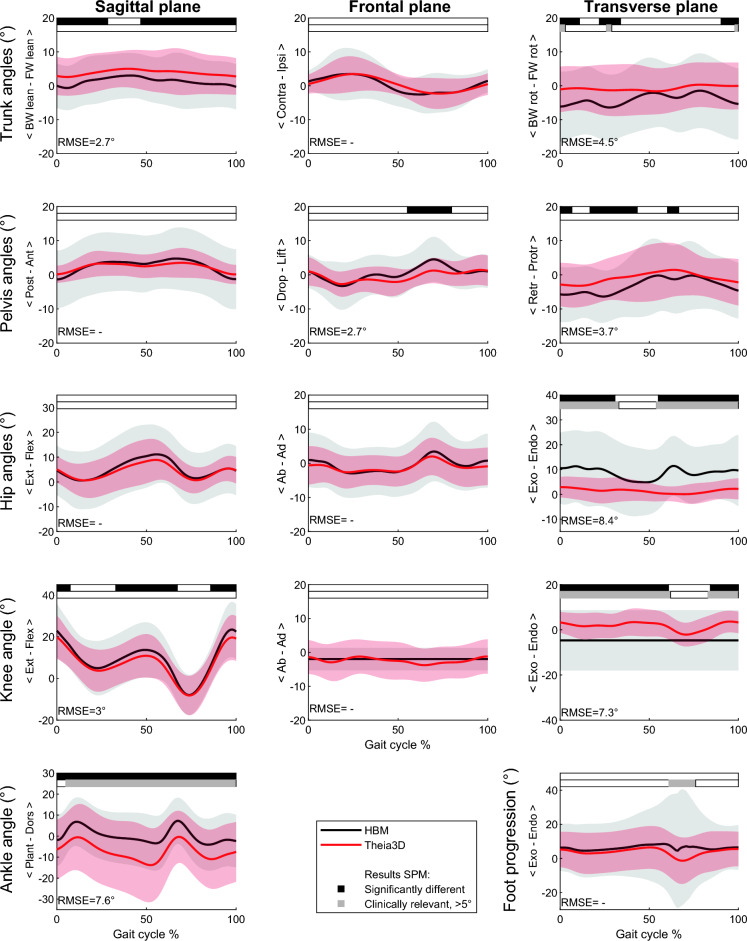
CP deviation from the norm—kinematic output (group mean ± SD) of the marker-based (HBM) and markerless (Theia3D) measurement systems. SPM outputs are displayed in the bars above each figure (white = non-significant, black = significant, grey = clinically relevant difference, > 5°). The RMSE of the significantly different parts of the curves are presented in the bottom left corner of each subplot.

When looking at the deviation from the norm (Fig. 4), the kinematic curves showed no systematic differences between the two systems, with exception of the decreased hip endorotation (RMSE of 8.4°), knee transverse rotation (RMSE of 7.3°) and increased ankle plantarflexion (RMSE of 7.6°) for the markerless system over almost the entire gait cycle.

In both TD children and children with CP, less inter-subject variability (as expressed by the SD) was present in the markerless system compared to the marker-based system for most kinematic variables (Table 1). Only for the ankle sagittal plane movement and knee ab-adduction in children with CP, the markerless system captured more variability compared to the marker-based system. The MAD between systems differed considerably between individuals, showing a large range in both groups (Table 1), with MAD ranging from a minimum difference of 0.8° as found for trunk lateroflexion and knee ab-adduction to a maximum of 45.3° observed in transverse hip rotation.

**Table 1:** The group mean standard deviations (SD) throughout the gait cycle per kinematic variable for HBM and Theia3D for TD children and children with CP; as well as the group mean absolute differences (MAD) and their range [min—max] between measurement systems per kinematic variable of the entire kinematic curves determined for TD and CP per child and averaged across children.

Angles	TD children					Children with CP			
	SD HBM (°)	SD Theia3D (°)	MAD (°)		MAD range	SD HBM (°)	SD Theia3D (°)	MAD (°)	MAD range
Trunk tilt	5.3	3.3	9.2	[1.0–18.0]		7.0	5.9	11.7	[3.6–19.0]
Trunk lateroflex	2.3	1.8	1.5	[0.8–2.5]		5.4	4.0	3.0	[0.8–13.6]
Trunk rotation	5.5	2.9	3.7	[1.8–8.5]		10.2	6.9	6.7	[1.4–20.2]
Pelvic tilt	4.5	2.3	8.9	[1.3–14.1]		8.6	3.5	9.6	[1.1–21.5]
Pelvic obliquity	2.5	1.8	2.5	[1.0–4.2]		5.7	3.7	2.9	[0.9–8.0]
Pelvic rotation	3.5	3.2	2.3	[1.3–4.7]		8.8	6.8	4.0	[1.0–8.2]
Hip flex/ext	5.5	3.3	7.0	[0.9–16.7]		11.2	6.7	8.9	[1.5–23.2]
Hip ab/ad	2.8	2.0	3.0	[1.6–4.7]		7.6	5.0	4.2	[1.0–8.9]
Hip rotation	5.9	2.5	7.3	[3.7–13.5]		13.2	4.1	12.7	[3.7–45.3]
Knee flex/ext	5.9	3.8	3.3	[1.6–5.5]		11.5	9.3	2.8	[1.0–11.6]
Knee ab/ad	2.6	1.9	5.2	[1.2–11.9]		3.3	5.1	6.8	[0.8–16.7]
Knee rotation	6.7	3.6	6.8	[2.3–17.4]		13.4	5.5	12.4	[1.2–35.3]
Ankle flex/ext	4.2	2.8	3.2	[1.3–4.9]		12.1	15.3	6.5	[0.9–32.7]
Foot progression	8.7	6.4	3.9	[1.6–9.0]		19.3	12.0	7.1	[1.1–30.2]

## Discussion

This study aimed to compare gait kinematics measured with a markerless and a marker-based motion capture system in TD children and children with CP. Systematic offsets were found in the sagittal plane of the trunk, pelvis and hip, and for knee ab-adduction and transverse hip and knee rotation. Additionally, in children with CP the markerless system systematically measured more plantarflexion. Looking at the deviation from the norm, differences remained only for transverse hip and knee rotation and ankle flexion. Moreover, inter-subject variability was lower for the markerless system compared to the marker-based system, while within-subject differences between systems varied largely between individuals.

The systematic offsets between systems as found in the sagittal plane for trunk, pelvis and hip kinematics and knee ab-adduction, for both TD and CP, were similar to previous studies in healthy adults^21^ and pediatric patients^23,25^. Differences in kinematic output between the two systems can partly be explained by differences in underlying model definitions. Specifically, the definitions of the anatomical segment coordinate systems and modeled joints differed between the two systems, which automatically lead to a systematic offset. Such systematic kinematic offsets between systems do not necessarily pose a problem clinically. In clinical practice, there is no comparison between systems, but rather patients are compared to normative data obtained using the same system and biomechanical definitions. In other words, it is more clinically relevant to establish whether the markerless system can accurately model motion which deviates from normative data and to compare this capability with the marker-based method, because that is the information that is used by clinicians to make their treatment decisions^7,27^. Hence, we used the deviation from the norm in this study as an outcome measure. This approach strongly reduced the systematic offsets between the systems, indicating that the offsets in the sagittal plane are partially explained by model definitions rather than tracking method.

Differences in transverse hip rotation between systems persisted when comparing to the norm data. Literature shows similar deviations in the transverse hip rotation between the two systems for abled adults^21^ and pediatric patients^23,25^. It is a well-known challenge to accurately measure transverse hip rotation with a marker-based system^28^, mainly due to soft tissue artifacts^17,32^. Hence, it is not evident which of the two systems measures the transverse hip rotation more accurately. However, since the markerless system hardly measured any rotation in the hip over the gait cycle for any of the subjects, and a high individual subject-level difference was present between systems, it can be questioned whether the markerless system is capable of capturing transverse hip rotation any more accurately than the marker-based system. In children with CP, increased hip endorotation is a common gait feature^8,27^, and therefore accurate tracking of transverse hip rotation is highly relevant in clinical practice. The marker-based system seems to better reflect those clinical observations (Fig. 3), so it might be that the marker-based system is modeling hip rotation more accurately than the markerless system, although no hard evidence can be given by this study. In addition to the transverse hip rotation, also knee transverse rotation remained different after comparing to the norm data. However, knee frontal and transverse angles should be compared with caution, as the HBM model only has a one DOF knee, whereas the Theia3D knee consists of 3 DOF.

Similarly, the markerless system appeared to have issues capturing individual variation in pelvic tilt in children with CP. When comparing the systems’ output to their own norm, no systematic kinematic offset was present anymore at group level. However, the markerless system did not capture the large variation in pelvic tilt in CP as measured by the marker-based system, as shown by the low SD for the markerless system. Part of the large variation in the marker-based system may be due to measurement error such as variation in marker placement and soft tissue artifact. However, the individual variation, up to 21 degrees in MAD for children with CP, is much larger than the 4 degrees as reported for reliability of pelvic tilt by Mc Ginley and colleagues with a marker-based system^31^. Hence, these findings seem to indicate that increased pelvic tilt within children with CP was not captured well with markerless motion capture.

This study is the first to show differences between markerless and marker-based systems in the ankle plantar/dorsiflexion, which was only present in CP. The markerless system measured systematically more ankle plantarflexion over the whole gait cycle. In contrast to the transverse hip rotation and pelvic tilt, the markerless system measured larger variations in the ankle compared with the marker-based system. Within the Theia3D software the foot consists of two segments and the foot and toes are tracked separately, while in the marker-based model (HBM) the complete foot is considered one rigid segment. It has been shown that tracking the hindfoot separately provides different ankle flexion angles (i.e. plantarflexion offset), especially in children with cerebral palsy^33^. It is known that children in CP often walk with increased plantar flexion in the ankle, and more dorsal flexion in the forefoot (i.e. a midfoot break)^8,27,34^. Hence, the larger variation measured by the markerless system might indicate that it is better capable of tracking the hindfoot than a marker-based mono-segment foot model, resulting in better tracking of the ankle joint itself instead of the whole foot. Future studies comparing both systems could include a marker-based multi-segment foot model for a more accurate comparison of the ankle angles.

Whether markerless motion capture can be implemented in clinical gait analysis, depends on the specific application. If the patient’s need is focused on pelvic tilt or transverse hip rotation, markerless motion capture is currently not recommended. Furthermore, especially in children with bony deformities, it is not recommended to use markerless motion capture, because in marker-based motion capture bony landmarks can be located by palpation and more accurate tracking is possible compared to solely video images. However, markerless motion capture can be applicable for issues concentrating on the knee and ankle in the sagittal and frontal plane. Furthermore, markerless motion capture may be a good and user-friendly alternative for marker-based motion capture in TD children, since increased pelvic tilt and transverse hip rotation are typically not an issue in these children. In any case, the systematical offsets in the sagittal plane for hip, pelvis and trunk need to be taken into account. Hence, it is recommended to gather a specific markerless normative dataset, to correct for this offset.

To further improve markerless motion capture for clinical applications, further training of the underlying neural network may be needed. Deep learning-based pose estimation as used in the markerless motion capture system is accurate at identifying anatomical landmarks from images it has been trained on, but it performs poorly at generalizing this estimation to movements and people with insufficient representation^35,36^. Populations with gait abnormalities and possible bony deformities as present in CP, may be underrepresented in the training image databases used for Theia3D, leading to the observed difficulties in tracking extreme angles. Therefore, it is highly recommended to include patient populations such as CP in the training data sets of markerless capture software, to facilitate clinical implementation of the system.

There are several limitations to the current study. First, we only evaluated the kinematics of barefoot walking. More types of data (e.g. kinetics) and gait conditions (for e.g. walking with shoes or orthoses) need to be studied to gain more insight into the potential of this method for clinical implementation. Second, we compared our markerless data only to the HBM marker model. The clinical use of the HBM model is yet limited and the model is mainly developed for real time applications on an instrumented treadmill. Besides the earlier discussed one DOF knee, the ankle joint between the two models could not be properly compared due to a differently modelled ankle joint. The markerless motion system could also be compared to other marker models, such as the Conventional Gait Model (CGM)^14^, CGM2^37^, or the Calibrated Anatomical System Technique (CAST) marker model^15^, because these have fewer joint constraints than HBM and may be able to measure rotations in the transverse plane in more detail. It is important to note however, that in a comparison study of Flux et al. (2020), much smaller and also more systematic differences were found between HBM and CGM or CAST, than we found here between HBM and Theia3D. Therefore, we do not expect the use of a different marker-based model to affect our main conclusions, as the effects seen here are of a different order than those observed between marker-based models themselves^28^. Another limitation of this study is that we could not perform a fulfilling correction of the differences in model definitions between systems, because that would require the exact definitions of the coordinate systems used by Theia3D, which remain company secret. Finally, it should be noted that skin-mounted retroreflective markers could influence the Theia3D tracking of anatomical features, as these were not present in the training dataset. Therefore, anatomical features that may be covered with markers may be more difficult to recognize and track by the Theia3D algorithm.

In conclusion, markerless motion capture was able to track frontal plane angles and sagittal plane knee and ankle angles well, but individual deviations in pelvic tilt and transverse hip rotation in CP as measured by the marker-based system, were not captured by the markerless system. Further training of the underlying deep learning network using images of pathological populations may help to improve the tracking of deviating gait features, allowing for a broader application of this new technology for clinical applications.

## Data Availability

The data that support the findings of this study are not openly available due to reasons of sensitivity and are available from the corresponding author upon reasonable request.
